# A Case of Term Delivery After Cervical Cerclage Following Hysteroscopic and Laparoscopic Uterine Scar Repair for Cesarean Scar Disorder

**DOI:** 10.1111/jog.70239

**Published:** 2026-03-08

**Authors:** Daiki Hiratsuka, Tomoko Makabe, Mitsunori Matsuo, Takayuki Iriyama, Miyuki Harada, Yasushi Hirota

**Affiliations:** ^1^ Department of Obstetrics and Gynecology, Graduate School of Medicine The University of Tokyo Tokyo Japan

**Keywords:** cervical cerclage, cesarean scar disorder, cesarean scar repair, cesarean section, infertility

## Abstract

A 33‐year‐old woman with secondary infertility after a term emergency cesarean delivery due to cesarean scar disorder (CSDi) underwent combined hysteroscopic and laparoscopic cesarean scar repair. Eight months later, at 34 years of age, she conceived spontaneously. Cervical length was 28.3 mm in early pregnancy (unchanged from preconception) but shortened to 20.5 mm at 13 + 5 weeks of gestation, and cervical insufficiency was suspected. A Shirodkar cerclage was performed at 15 + 4 weeks of gestation. Thereafter, cervical shortening did not progress and the pregnancy remained uncomplicated. Elective cesarean delivery at 37 + 1 weeks of gestation resulted in a healthy female infant, and both maternal and neonatal courses were uneventful. Cesarean scar repair may affect cervical competence; thus, pregnancies after CSDi repair should include close cervical length surveillance and consideration of cerclage when significant shortening is detected. Further evidence is needed to optimize postoperative pregnancy management.

**Abbreviations**
AUB: abnormal uterine bleeding
CSDi: cesarean scar disorder
MRI: magnetic resonance imaging
RMT: residual myometrial thickness

## 1 Introduction

Cesarean scar disorder (CSDi) is a condition characterized by thinning and fluid accumulation at the site of the previous cesarean section incision in the uterine myometrium, leading to abnormal uterine bleeding (AUB), dysmenorrhea, and secondary infertility [1]. With the global rise in cesarean deliveries, cesarean scar defects have become increasingly common, occurring in up to 60% of women after cesarean section [2]. Among these, approximately 30%–40% develop symptoms, making this one of the most prevalent and clinically significant disorders among women of reproductive age [2, 3]. In particular, for women presenting with secondary infertility caused by CSDi, hormonal therapy is ineffective, and surgical repair of the cesarean scar—performed hysteroscopically, laparoscopically, or in combination—remains the mainstay of treatment [3, 4].

Minimally invasive surgery is an important treatment option for patients who wish to conceive [5, 6], and cesarean scar repair has been shown to restore the uterine environment and improve fertility outcomes [7]. As these surgical approaches become more widespread, pregnancies after CSDi repair are expected to increase. However, some recent studies have also raised concerns about potential obstetric complications during such pregnancies, including cervical shortening and preterm birth, possibly related to altered uterocervical anatomy and physiology following surgical intervention [8, 9]. Despite these observations, preventive strategies for cervical shortening or preterm delivery following surgical repair of a cesarean scar remain lacking.

We herein report a rare case of a woman with secondary infertility due to CSDi who conceived spontaneously after combined hysteroscopic and laparoscopic cesarean scar repair. At 13 weeks of gestation, significant cervical shortening was detected, leading to the performance of a therapeutic Shirodkar cervical cerclage. The pregnancy progressed uneventfully, and a healthy infant was delivered at 37 weeks of gestation by elective cesarean section. This case highlights the potential reproductive benefits of scar repair and illustrates a successful management strategy for cervical insufficiency following cesarean scar repair.

## 2 Case Presentation

A 33‐year‐old woman, gravida 2 para 1, presented with a 2‐year history of secondary infertility as her chief complaint, accompanied by AUB characterized by persistent brown spotting lasting for approximately 10 days after menstruation and increased vaginal discharge. An initial infertility work‐up revealed no other identifiable causes.

Her obstetric history was notable for a spontaneous conception followed by an emergency cesarean section at 41 weeks of gestation for non‐reassuring fetal status during the latent phase at age 29. The uterine incision was closed with a double‐layer myometrial suture. That pregnancy was not complicated by cervical shortening or threatened preterm labor.

**FIGURE 1:**
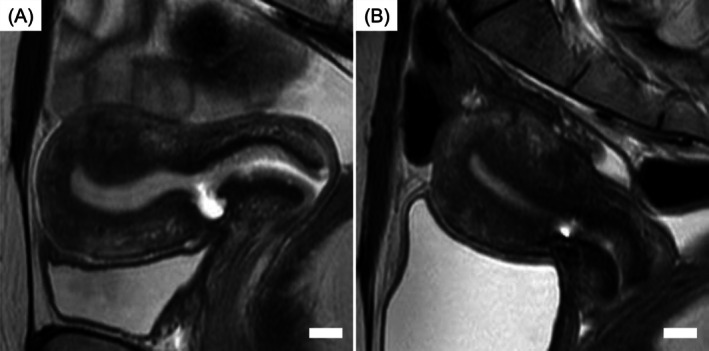
T2‐weighted sagittal MRI findings of the uterus before pregnancy. (A) MRI before cesarean scar repair. A cesarean scar niche measuring 8.1 mm in depth and 6.7 mm in width was observed. The RMT was 1.8 mm, and the cervical length measured 36.8 mm. (B) MRI after cesarean scar repair. The RMT increased to 6.5 mm, and the cervical length measured 28.3 mm. The white bars indicate 10 mm. MRI, magnetic resonance imaging; RMT, residual myometrial thickness.

Within 2 months of her initial visit, a diagnostic work‐up was performed. Transvaginal ultrasonography, magnetic resonance imaging (MRI), and hysteroscopy were performed to assess the scar defect and residual myometrial thickness (RMT), delineate pelvic anatomy and coexisting uterine pathology, and directly visualize the niche in the cervico‐isthmic region. These investigations demonstrated a retroflexed uterus with marked thinning of the anterior myometrium at the prior cesarean incision. The RMT was 1.8 mm, and a fluid‐filled pouch measuring 8.1 mm in depth and 6.7 mm in width was observed at the same site (Figure 1A). Hysteroscopy revealed a fluid‐filled reservoir near the internal os and significant thinning of the anterior uterine wall at the cesarean scar site, where scarred endometrium and abnormal vascularity were observed. Based on these findings and symptoms, CSDi was diagnosed [1]. Preoperatively, the cervical length measured 36.8 mm. Given the markedly thin RMT and the need for anatomical reconstruction, hysteroscopic treatment alone was considered insufficient; therefore, a combined hysteroscopic and laparoscopic approach was planned.

**FIGURE 2:**
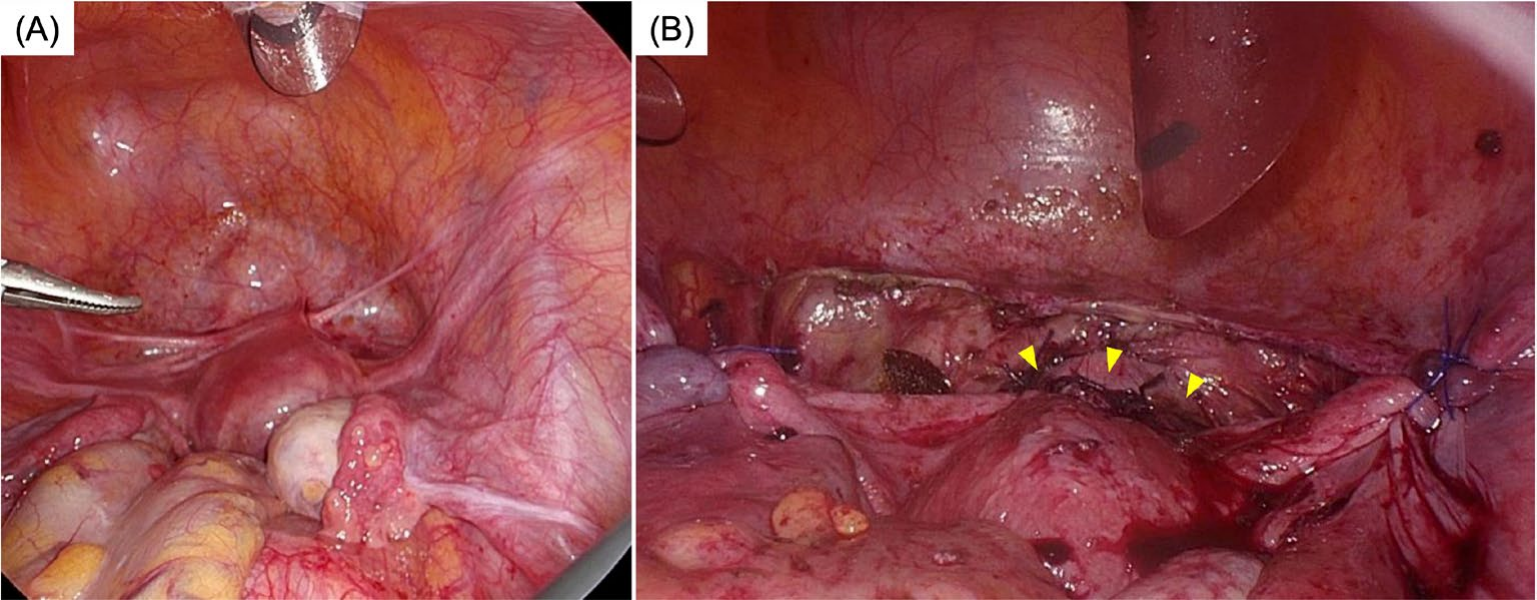
Intraoperative findings. (A) Before repair (at the start of surgery). Peritoneal traction in the vesicouterine pouch was observed. (B) After myometrial repair. The myometrium was reconstructed and bilateral round ligament plication was performed; the vesicouterine peritoneum was subsequently sutured. The yellow arrowheads indicate the myometrial suture line.

Three months after her initial visit, combined hysteroscopic and laparoscopic cesarean scar repair was performed. Intraoperative findings revealed peritoneal traction and fibrous adhesions in the vesicouterine pouch, consistent with postoperative changes following cesarean section. The uterus was normal in size, with mildly lax round ligaments, and was retroflexed. At the site of the cesarean scar, the myometrium was markedly thinned to the extent that the underlying layers were visible. Additionally, a subcentimeter peritoneal endometriotic lesion was identified on the uterine serosa, corresponding to an rASRM score of 1 (Stage I). The surgical procedure began with hysteroscopic resection of the cesarean scar tissue, focusing on removing the cranial and caudal portions of the defect. This was followed by laparoscopic surgery to excise the remaining scar tissue, after which the uterine wall was repaired with a two‐layer laparoscopic suture. Bilateral round ligament plication was also performed to correct uterine retroflexion. Representative intraoperative findings are shown in Figure 2.

At 3 months postoperatively, MRI and hysteroscopic evaluation demonstrated satisfactory healing of the uterine scar without abnormal vascularity, with the RMT improved to 6.5 mm (Figure 1B). Cervical length at that time was 28.3 mm, and pregnancy was permitted.

**FIGURE 3:**
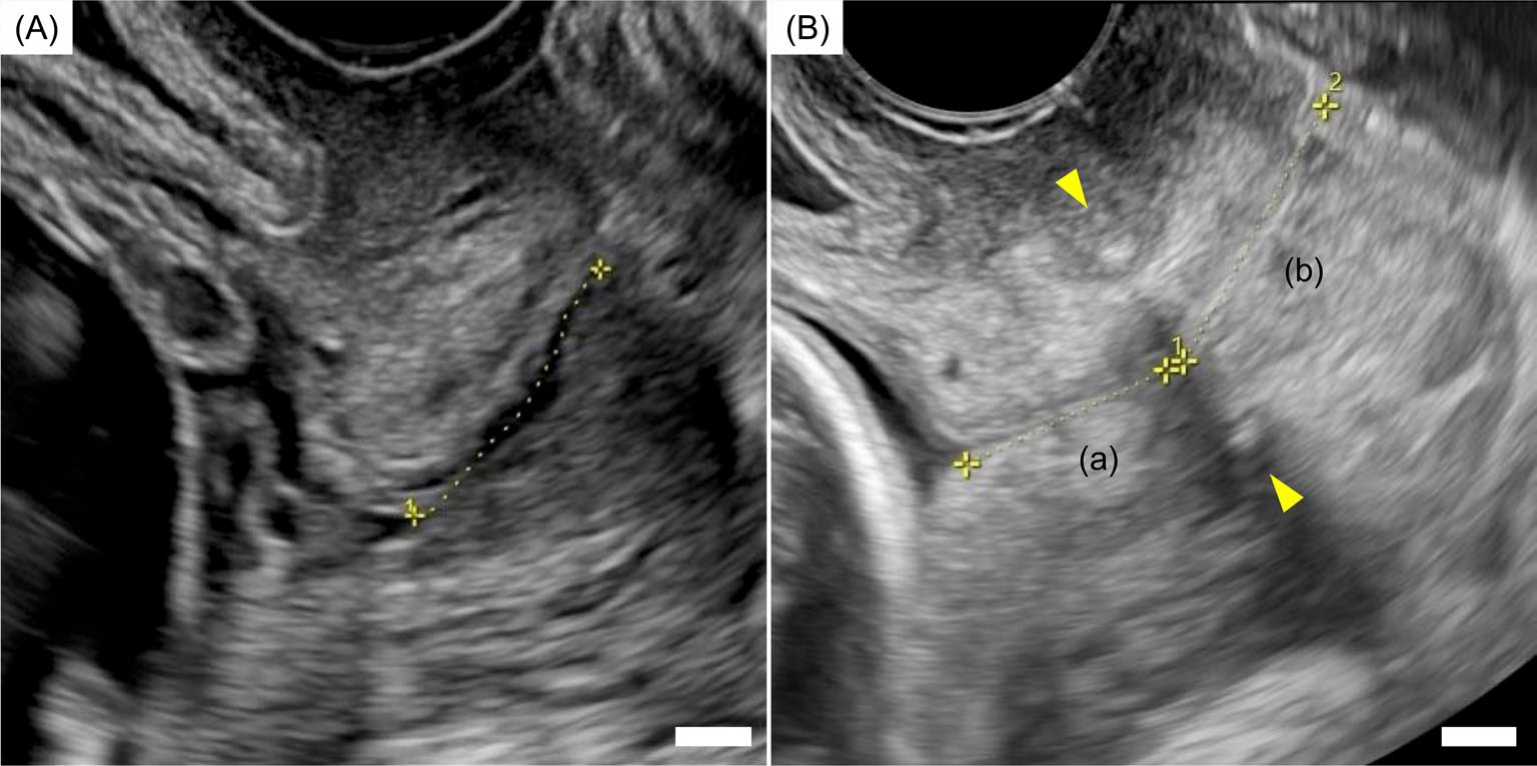
Transvaginal ultrasonography findings during pregnancy. (A) Transvaginal ultrasonography before cervical cerclage. The yellow line represents the total cervical length, measured at 20.5 mm. (B) Transvaginal ultrasonography after cervical cerclage. The yellow line labeled “(a)” indicates the distance from the internal os to the Shirodkar cerclage tape (14.7 mm), while the line labeled “(b)” indicates the distance from the external os to the cerclage tape (19.5 mm). The yellow arrowheads indicate the location of the Shirodkar cerclage tape. The white bars indicate 5 mm.

AUB resolved, and the patient conceived spontaneously 8 months postoperatively, at age 34. In early pregnancy, the cervical length remained 28.3 mm. However, at 13 weeks + 5 days of gestation, the cervical length had shortened to 20.5 mm, with wedge‐shaped internal os funneling (Figure 3A). She reported no abdominal pain or uterine contractions, and there were no clinical signs suggestive of infection. Suspected cervical insufficiency was considered given the early progressive cervical shortening with internal os funneling. At 15 weeks + 4 days of gestation, a Shirodkar cervical cerclage was performed (Figure 3B). Postoperatively, the distance from the internal os to the cerclage tape was 14.7 mm, and from the cerclage to the external os was 19.5 mm. Cervical shortening did not progress thereafter, and the pregnancy continued uneventfully.

At 37 weeks + 1 day of gestation, an elective cesarean section was performed, resulting in the delivery of a healthy female infant weighing 2852 g. Intraoperatively, the lower uterine segment was markedly thinned, and the fetal head was visible through the uterine wall; no myometrial thinning was observed in the posterior uterine wall. Both the maternal and neonatal postoperative courses were uneventful.

## 3 Discussion

This case describes a rare and valuable example in which hysteroscopic and laparoscopic uterine scar repair for CSDi successfully improved secondary infertility, leading to spontaneous conception, followed by therapeutic cervical cerclage and term delivery. To the best of our knowledge, reports of cervical cerclage performed during pregnancy after surgical repair for CSDi are scarce.

The prevalence of CSDi has been increasing in parallel with the global rise in cesarean section rates, highlighting its growing clinical significance [2, 3]. Until recently, the diagnostic criteria for CSDi were not standardized, making it difficult to conduct homogeneous analyses across studies. However, the condition was formally defined in 2023 [1], emphasizing the urgent need to accumulate clinical data and establish evidence‐based management strategies to improve the care of affected women.

For patients presenting with secondary infertility due to CSDi, surgical repair is often effective [7]; however, comprehensive management strategies, including postoperative pregnancy surveillance, remain limited. CSDi repair techniques are most commonly performed using hysteroscopy and/or laparoscopy. Hysteroscopic resection, which involves shaving and coagulating the scar niche, is a minimally invasive procedure but does not achieve anatomical restoration. This may enlarge the defect and increase the risk of uterine rupture in subsequent pregnancies. In contrast, laparoscopic repair allows complete excision of the scar tissue and anatomical reconstruction of the myometrium by layered suturing. Because the RMT in the present case was only 1.8 mm, laparoscopic repair was chosen. However, excessive resection of normal myometrium surrounding the scar—particularly near the cervical region—during laparoscopic surgery may increase the risk of preterm birth due to postoperative cervical shortening. Therefore, in this case, hysteroscopy was combined with laparoscopy to precisely delineate the lesion and limit excision to the minimum extent necessary, resulting in successful repair with an RMT of 6.5 mm [10]. Although mild cervical shortening was observed postoperatively in this case, advances in surgical planning and technique selection have contributed to higher rates of successful repair without compromising cervical length [10].

Reports on preterm labor following laparoscopic repair of CSDi are limited. Some studies have reported preterm birth rates comparable to those in normal pregnancies [11], whereas others have described much higher rates [8, 9]. Thus, continued vigilance regarding preterm delivery after CSDi repair is warranted. Currently, no consensus exists regarding cervical shortening or the role of cerclage in pregnancies after CSDi repair. In the present case, cervical length was 28.3 mm before conception and in early pregnancy, but soon shortened to 20.5 mm at 13 weeks of gestation. Because physiologic shortening is not typically observed in early to mid‐pregnancy [12], and cervical shortening was not observed during her previous term pregnancy, the cervical change in the present case was more consistent with a pathological process and may have been related to the preceding CSDi repair. A Shirodkar cervical cerclage was therefore performed, which stabilized the cervical length and resulted in a term delivery. These findings suggest that, while advances in CSDi repair techniques should aim to minimize pre‐existing cervical shortening [10], careful monitoring of cervical length during pregnancy after scar repair remains crucial. When non‐physiological cervical shortening is detected, therapeutic cervical cerclage may be a useful management option. Furthermore, reports exist of postoperative cervical stenosis developing months after surgery, implying that mechanisms of cervical dysfunction may extend beyond simple shortening [13]. In addition, a potential association between CSDi and chronic endometritis—a condition recognized as an important cause of infertility owing to its impact on endometrial receptivity—has also been reported [14, 15]. In this context, the presence or absence of chronic inflammatory changes in the cervix or around the repaired uterine scar may influence the subsequent course of pregnancy after CSDi surgery. Therefore, evaluating local inflammation in the cervico‐isthmic region could be an important component of postoperative assessment and management.

Hence, further accumulation of well‐characterized cases and long‐term follow‐up studies is needed to clarify the relationship between CSDi repair, cervical function, and pregnancy outcomes.

In conclusion, we experienced a case in which combined hysteroscopic and laparoscopic repair for CSDi restored the uterine environment and enabled spontaneous conception, and in which a term delivery was successfully achieved following therapeutic cervical cerclage. This case suggests that cesarean scar repair may influence cervical competence during subsequent pregnancies. Therefore, pregnancies after CSDi repair should involve close surveillance of cervical length, and cerclage should be considered when significant shortening is identified. As pregnancies after CSDi repair are expected to increase, the accumulation of clinical evidence regarding postoperative pregnancy management will be essential for optimizing outcomes.

## Author Contributions

**Daiki Hiratsuka:** conceptualization, data curation, writing – original draft, investigation, methodology, formal analysis. **Tomoko Makabe:** review and critical comments. **Mitsunori Matsuo:** data curation, review and critical comments. **Takayuki Iriyama:** data curation, formal analysis. **Miyuki Harada:** review and critical comments. **Yasushi Hirota:** funding, writing – review and editing.

## Funding

This work was supported by Japan Society for the Promotion of Science, JP23K15827, JP24K23524, JP23K27176, JP23K24481, JP24K22157, JP23K23803, JP24K21911; Japan Agency for Medical Research and Development, JP24gn0110085, JP24gn0110069, JP24gk0210039, JP24lk0310083; Japan Science and Technology Agency, JPMJFR210H; Children and Families Agency, JPMH23DB0101.

## Disclosure

The authors have nothing to report.

## Ethics Statement

The authors have nothing to report.

## Consent

Written informed consent was obtained from the patient for publication of this case report.

## Conflicts of Interest

Dr. Yasushi Hirota and Dr. Takayuki Iriyama are Editorial Board members of this submitted JOGR Journal and co‐authors of this article. To minimize bias, they were excluded from all editorial decision‐making related to the acceptance of this article for publication.

## Data Availability

Data sharing not applicable to this article as no datasets were generated or analyzed during the current study.
